# RET-independent signaling by GDNF ligands and GFRα receptors

**DOI:** 10.1007/s00441-020-03261-2

**Published:** 2020-07-31

**Authors:** Carlos F. Ibáñez, Gustavo Paratcha, Fernanda Ledda

**Affiliations:** 1 Department of Neuroscience, Karolinska Institute, 17177 Stockholm, Sweden; 2 Department of Physiology, National University of Singapore, Singapore, 117597 Singapore; 3 Life Sciences Institute, National University of Singapore, Singapore, 117456 Singapore; 4 Instituto de Biología Celular y Neurociencias, Universidad de Buenos Aires, CONICET, Buenos Aires, Argentina; 5 Fundación Instituto Leloir, Instituto de Investigaciones Bioquímicas de Buenos Aires, CONICET, Buenos Aires, Argentina

**Keywords:** Neurodevelopment, Cell migration, Axon guidance, Synaptogenesis

## Abstract

The discovery in the late 1990s of the partnership between the RET receptor tyrosine kinase and the GFRα family of GPI-anchored co-receptors as mediators of the effects of GDNF family ligands galvanized the field of neurotrophic factors, firmly establishing a new molecular framework besides the ubiquitous neurotrophins. Soon after, however, it was realized that many neurons and brain areas expressed GFRα receptors without expressing RET. These observations led to the formulation of two new concepts in GDNF family signaling, namely, the non-cell-autonomous functions of GFRα molecules, so-called *trans* signaling, as well as cell-autonomous functions mediated by signaling receptors distinct from RET, which became known as RET-independent signaling. To date, the best studied RET-independent signaling pathway for GDNF family ligands involves the neural cell adhesion molecule NCAM and its association with GFRα co-receptors. Among the many functions attributed to this signaling system are neuronal migration, neurite outgrowth, dendrite branching, spine formation, and synaptogenesis. This review summarizes our current understanding of this and other mechanisms of RET-independent signaling by GDNF family ligands and GFRα receptors, as well as their physiological importance.

## Introduction

Although the concept of neurotrophic factors as target-derived survival molecules originated from studies of the developing nervous system, it quickly grabbed the attention of researchers investigating neuronal death in neurodegenerative diseases, such as Parkinson’s disease, and how to prevent it. The early 1990s saw the expansion of the neurotrophin family, the paradigm of neurotrophic factors, and a flurry of activity characterizing their functions in different neuronal populations. However, the neurotrophins were never very potent at promoting survival of midbrain dopaminergic neurons, the main neuronal population that degenerates in Parkinson’s disease, and the search was on at multiple laboratories for what many scientists thought would be “bonafide” survival factors for this class of neurons. This is how glial cell line-derived neurotrophic factor (GDNF) was discovered and first reported in 1993 (Lin et al. 1993). Structural similarities between GDNF and members of the TGFβ superfamily initially misdirected efforts to identify GDNF receptors. The RET receptor tyrosine kinase was eventually identified as the first functional receptor for GDNF, based on its biochemical properties as well as the phenotypic similarities between RET and GDNF knock-out mice (Durbec et al. 1996; Trupp et al. 1996). But RET could not bind GDNF on its own with high affinity, and so expression-cloning studies based on GDNF binding led to the identification of the GPI (glycosyl-phosphatidyl-inositol)-anchored co-receptor GFRα1 (named GDNFR-α at the time) as a necessary component of the GDNF receptor complex together with RET (Jing et al. 1996; Treanor et al. 1996). The concept was attractive in its elegance and simplicity. RET could not bind GDNF with high affinity, but it could signal through its tyrosine kinase domain. And while GFRα1 could bind GDNF, it was not expected to signal due to its lack of an intracellular domain. This notion required strict co-expression of the two receptors in the same cell for a functional complex to be assembled, and, indeed, midbrain dopaminergic neurons do express both RET and GFRα1 (Treanor et al. 1996). However, several detailed expression studies that followed could show that many other neurons in the brain as well as cells elsewhere did not (Trupp et al. 1997; Yu et al. 1998). Although the subsequent discovery of GFRα family members GFRα2, 3, and 4, all of which associate with RET to mediate signaling by GDNF family ligands NTN, ART, and PSP, respectively (reviewed in Airaksinen and Saarma (2002), Airaksinen et al. (1999)) helped to account for sites that expressed RET without GFRα1, it only expanded the list of cell types that had GFRα receptors but not RET. What was going on in those cells?

At the time, a small group of researchers adhered to the null hypothesis, namely, that GFRα molecules were actually not doing anything on cells that did not express RET, an argument that echoed Stephen J. Gould’s famous spandrels in San Marco’s Cathedral (Gould and Lewontin 1979). In fact, some evidence was presented which could be said supported that notion (Enomoto et al. 2004). Admittedly, a lot remained to be discovered back then. Other researchers, however, held on to the traditional aphorism that “absence of evidence is not evidence of absence” and so rationalized the problem of GFRα expression without RET along the only two logical, though non-mutually exclusive, possibilities available, namely, cell-autonomous and non-cell-autonomous functions. In the latter scenario, GFRα molecules were proposed to function in *trans* by presenting GDNF ligands to RET receptors expressed on other cells. In the former, GFRα receptors were envisioned to function without RET cell autonomously, either mediating signaling on their own or together with other co-receptors, a concept which became known as RET-independent signaling. In this review, we first discuss mechanisms of *trans* signaling in brief form, as these mainly utilize RET and so do not formally fall into RET-independent signaling. This is followed by a presentation of the phenomenon of ligand-induced cell adhesion-mediated GDNF and GFRα molecules independently of RET. We then present a more extended description of RET-independent pathways, expanding on NCAM-mediated signaling as well as other evidence pointing to alternative co-receptors and mechanisms.

### Signaling in *trans* by GFRα molecules

GPI-anchored molecules lack transmembrane and intracellular domains; they are attached to the outer leaflet of the plasma membrane by a glycolipid link. They are constitutively shed from the surface of cells through the action of cell surface lipases. It has been shown that GFRα1 can be released from expressing cells and accumulate in soluble form in the cell supernatant (Ledda et al. 2002; Paratcha et al. 2001). Soluble GFRα1 is bioactive in that it can bind GDNF and stimulate the activation of RET in cells (Paratcha et al. 2001; Treanor et al. 1996; Yu et al. 1998). Released GFRα1 has been proposed to mediate *trans* signaling by capturing GDNF and presenting it to RET receptors through at least three mechanisms: in soluble form, attached to the extracellular matrix or from the membrane of adjacent cells (Paratcha et al. 2001). Many GDNF-responsive neurons expressing RET project to brain regions rich in GFRα1 expression, suggesting that they may normally be exposed to GFRα1 molecules in *trans* (Trupp et al. 1997; Yu et al. 1998). The glycolipid moiety of GPI-anchored receptors has affinity for specialized regions of the plasma membrane known as lipid rafts (Simons and Toomre 2000), and GFRα1 readily partitions into these compartments (Paratcha et al. 2001; Tansey et al. 2000; Trupp et al. 1999). Elegant studies showed that GFRα1 can recruit RET to lipid rafts upon GDNF binding, allowing RET to signal from this membrane compartment (Paratcha et al. 2001; Tansey et al. 2000). Intriguingly, activated RET can trigger different signaling pathways depending on whether it is inside or outside rafts. For example, activated RET was shown to associate with and phosphorylate different adaptor proteins depending on its raft location: SHC outside lipid rafts and FRS2 inside rafts (Paratcha et al. 2001). Altering the location of active receptors in different membrane compartments regulates and diversifies intracellular signal transduction. As exogenous GFRα1 is not associated with lipid rafts, it was initially thought that activation in *trans* would be unable to direct RET to these membrane compartments (Tansey et al. 2000). But other studies could demonstrate that exogenous GFRα1 did indeed function in *trans* to relocate RET to lipid rafts, albeit with a slower kinetics (Paratcha et al. 2001). Interestingly, recruitment of RET to lipid rafts in *trans*, but not in *cis*, was found to depend upon its tyrosine kinase activity, as well as the phosphorylation of Tyr^1062^, the FRS2-binding site in RET (Paratcha et al. 2001). The FRS2 adaptor protein associates with lipid rafts through a saturated acyl chain (Kouhara et al. 1997), suggesting that lipid raft recruitment of RET in *trans* was regulated by an intracellular mechanism, probably through interaction with FRS2 (Paratcha et al. 2001). These results represented the first evidence that receptors can be compartmentalized at the plasma membrane by both extracellular and intracellular mechanisms (Paratcha and Ibáñez 2002). The in vivo physiological importance of lipid raft signaling by GFRα1 was probed in a study using transgenic mice expressing a *Gfra1* cDNA construct carrying a transmembrane domain in place of the GPI-anchored signal sequence introduced into the *Gfra1* locus by homologous recombination, thus disrupting the expression of the endogenous gene (Tsui et al. 2015). This construct had been produced in an earlier study and shown not to translocate to lipid rafts in a cell line model (Tansey et al. 2000). Although the knock-in mice expressed the transmembrane GFRα1 at normal levels, homozygous mice showed several phenotypes that are characteristic of null mice lacking GFRα1, including renal agenesis and loss of enteric neurons (Tsui et al. 2015). Surprisingly, a protein showing GFRα1 immunoreactivity was detected from supernatants of cells cultured from the knock-in mice, indicating that it could be shed similarly to its wild-type counterpart. However, whether the shed protein had any biological activity in *trans* was not tested in the study. It also remained unclear from these studies whether the transmembrane construct could still be present in the lipid rafts of the mouse neurons or glial cells, despite the earlier cell line result, as this was not assessed in the study. In any case, this work strongly suggests that lipid raft signaling by GFRα1 is also relevant in vivo.

It was later shown that GFRα signaling in *trans* offered different possibilities to enhance and localize RET activity in neurons as well as other cells. Worley et al. demonstrated that both GFRα1 and GFRα2 added exogenously to cultures of enteric neurons potentiated responses to GDNF and NTN, even in neurons that also expressed endogenous GFRα receptors (Worley et al. 2000). Intriguingly, they found that the specificity of the responses to exogenous GFRα molecules correlated with the type of cognate GFRα receptors expressed in *cis*. In another study, Ledda et al. investigated functions of exogenous GFRα1 molecules presented in localized fashion to growth cones and axons of sensory neurons by coating beads with recombinant GFRα1 protein (Ledda et al. 2002). Under those conditions, exogenous GFRα1 was found to potentiate neurite outgrowth and act as a long-range directional cue by creating positional information for RET-expressing axons in the presence of uniform concentrations of GDNF (Ledda et al. 2002). Immobilized GFRα1 was able to drive and reorient axonal growth along sites of GFRα1 expression. Mechanistically, exogenous GFRα1 enhanced and sustained the activation of cyclin-dependent kinase 5 (Cdk5), a lipid raft protein and well-known regulator of axonal growth and guidance (Dhavan and Tsai 2001). GFRα1 was the first receptor shown to act non-cell autonomously as a guidance cue for neurons (Crone and Lee 2002).

More recent studies from the past few years have shown that *trans* signaling by GFRα receptors continues to explain a multitude of biological processes. Patel and colleagues showed that, in the gut, lymphoid tissue initiator cells expressing RET responded to GDNF family ligands presented in *trans* by GFRα receptors expressed by adjacent cells (Patel et al. 2012). In contrast, RET could be activated in *cis* in enteric neurons (Patel et al. 2012). The multifaceted RET responses were determined by distinct patterns of expression of the genes encoding RET and its co-receptors. The authors concluded that activation of RET in *trans* was being deployed to control the initial phase of enteric lymphoid organ morphogenesis, indicating that the specificity of RET signaling can be regulated by differential co-expression of RET and GFRα receptors. In another study, He et al. showed that GFRα1 released by sensory nerves could enhance cancer cell perineural invasion through activation of RET signaling in *trans* (He et al. 2014). The authors found that RET activation, MAPK pathway activity, and cancer cell migration towards GDNF were increased upon exposure to soluble GFRα1. Intriguingly, human pancreatic ductal adenocarcinomas demonstrated great variability in their expression of GFRα1, indicating alternative sources of GFRα1 in perineural invasion by these cancer cells (He et al. 2014). One last example reviewed here is the recent work by Fleming and colleagues, showing how rapidly adapting mechanoreceptors in dorsal root ganglia utilize GFRα2 in *cis* and GFRα1 in *trans* to mediate RET activation necessary for survival and central projection growth (Fleming et al. 2015). They find that mice lacking RET show central projection deficits which were phenocopies by *Gfra1;Gfra2* double knock-outs, but not by the single knock-outs (Fleming et al. 2015). They demonstrated that GFRα1 produced by neighboring ganglion neurons activates RET in mechanoreceptors, suggesting that *trans* and *cis* RET signaling could function in the same developmental process. Together, these studies show that *trans* signaling is deployed in a number of developmental as well as physiological and pathophysiological processes to enhance and diversify RET signaling.

### RET-independent ligand-induced cell adhesion by GDNF and GFRα receptors

The discovery that GDNF could trigger trans-homophilic binding between GFRα1 molecules and cell adhesion between GFRα1-expressing cells, independently of RET, presented for the first time the phenomenon of “ligand-induced cell adhesion,” a previously unknown mechanism for regulated cell-cell interaction that combines features of both diffusible and membrane-bound signals (Ledda et al. 2007). Subsequently, examples of ligand-induced cell adhesion were found in other systems, such as the ability of cerebelins to mediate cell adhesion between cells expressing glutamate receptors and Neurexins (Uemura et al. 2010) and Slit-mediated cell adhesion between cells that express Robo and Neurexin (Blockus et al. 2019).

Conventional cell adhesion mechanisms involve interactions that occur by default, triggered by the simple encounter of cell adhesion molecules with their partners in either homophilic or heterophilic interactions. In contrast, ligand-induced cell adhesion is a mechanism for cell-cell interaction regulated by a soluble extracellular cue. The requirement for a soluble signal makes it very versatile and highly regulatable. In addition, by stimulating contact between two different cells, the ligand may also trigger interactions between other ligand-receptor systems and thereby indirectly elicit signaling events that could also contribute to development and physiology. The mechanisms by which GDNF can induce adhesion between cells expressing GFRα1 receptors are still unclear. It was initially suggested that GDNF could promote homophilic interactions between GFRα1 molecules in different cells by “trans-dimerization,” acting as a physical bridge between them. However, other ligands known to induce receptor dimerization, such as nerve growth factor, were shown to be incapable of inducing adhesion between receptor-expressing cells (Ledda et al. 2007). Another possibility is that GDNF acts through an allosteric mechanism by inducing conformational changes in GFRα1 that expose determinants responsible for trans-homophilic binding. Intriguingly, our unpublished results indicate that contacts induced by GDNF between GFRα1-expressing cells are dependent on extracellular heparin and the ability of GDNF and GFRα1 to bind heparin-like molecules, suggesting that heparin-like substances may help to bridge GDNF/GFRα1 complexes across cells during ligand-induced cell adhesion (Ibáñez et al., unpublished).

**Fig. 1:**
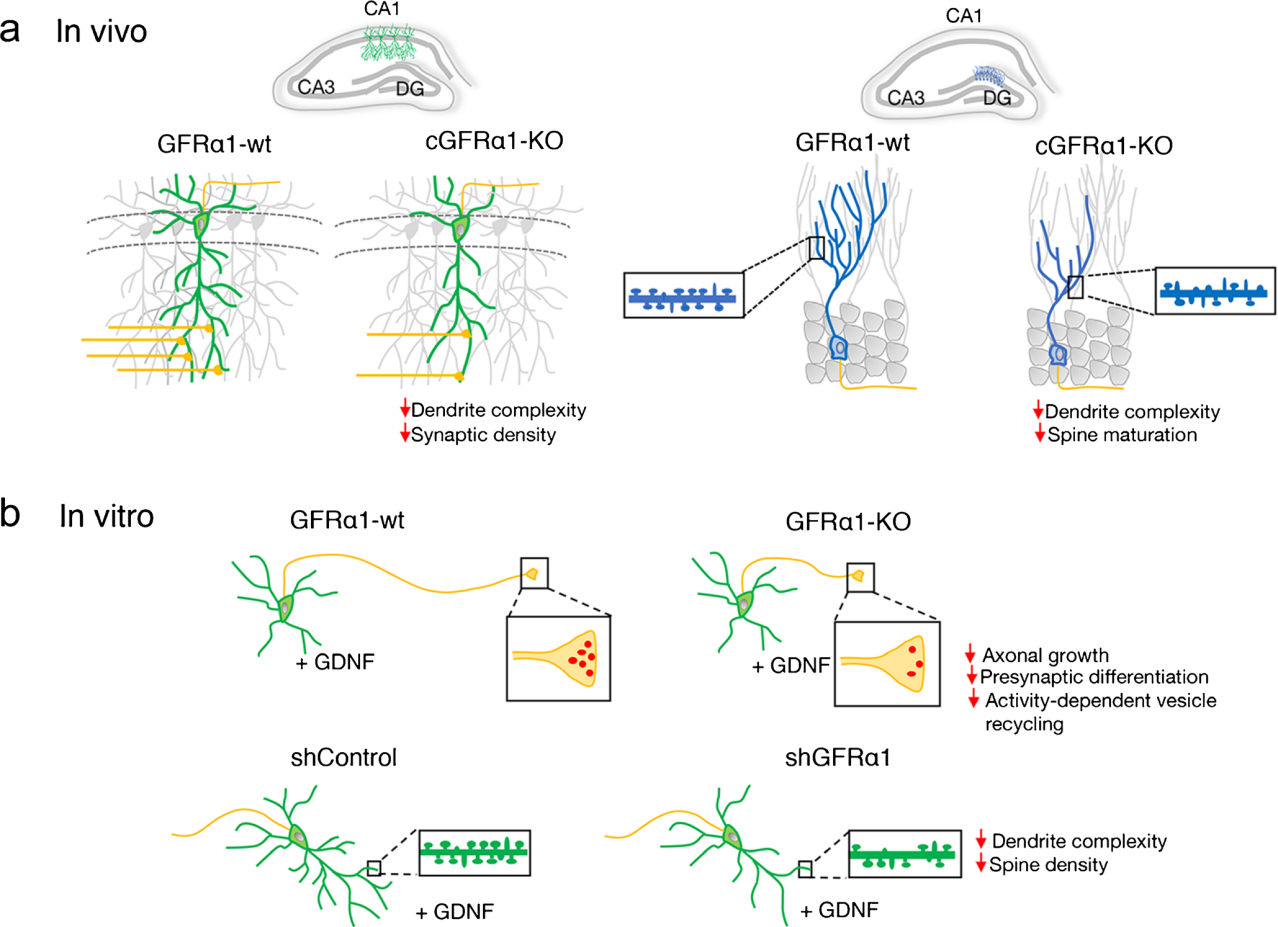
Schematic representation of the functional and structural plasticity promoted by GDNF/GFRα1 in hippocampal neurons. The upper panel (**a**) describes the morphological plastic changes reported in vivo in pyramidal (green) and adult-born dentate gyrus (DG) granule cells (GCs) (blue) from wild-type (GFRα1-wt) and conditional GFRα1 knock-out (cGFRα1-KO) mice. The lower panel (**b**) describes the axonal and dendritic effects induced by GDNF reported in hippocampal primary cultures in which GFRα1 expression was downregulated by knock-out (GFRα1-KO) or knock-down (shGFRα1). The inserts display examples of axonal terminals containing presynaptic vesicles and dendritic shafts showing an array of mushroom (mature) and thin/stubby (immature) dendritic spines. Axons are represented in yellow

A key physiological function for RET-independent ligand-induced cell adhesion by GDNF and GFRα receptors has been demonstrated in synapse formation and maintenance (reviewed in (Ledda 2007)). In the first publication by Ledda et al., it was shown that beads coated with GFRα1 could induce localized presynaptic differentiation in hippocampal neurons at the sites of contact with axons, as visualized by clustering of vesicular proteins and neurotransmitter transporters and by activity-dependent vesicle recycling (Ledda et al. 2007) (Fig. 1). This effect required the presence of GDNF in the culture medium, and in fact, GFRα1-coated beads adhered to the surface of hippocampal neurons only when GDNF was added. GFRα1 was found enriched at pre- and postsynaptic compartments in hippocampal neurons, and presynaptic differentiation induced by GDNF was markedly reduced in neurons lacking GFRα1. Mice that were heterozygous for a null mutation in the *Gdnf* gene showed reduced synaptic localization of presynaptic proteins and a marked decrease in the density of presynaptic puncta (Ledda et al. 2007). In agreement with the importance of GDNF and GFRα1 for synaptogenesis in the mouse hippocampus, a later study used conditional *Gfra1* knock-out mice lacking the receptor in subpopulations of forebrain neurons and astrocytes and found that GFRα1 is required for proper hippocampal dendritic arborization and spine formation in vivo (Irala et al. 2016) (Fig. 1). Hippocampal neurons lacking GFRα1 affected postsynaptic assembly, indicating that GFRα1 is a bidirectional synaptic organizing protein. These researchers found that signaling by the neural cell adhesion molecule NCAM was required for induction of dendrite growth and spine formation by GDNF/GFRα1 in their system (Irala et al. 2016). In the following section, we expand on the activities and physiological function of NCAM as an alternative, RET-independent, signaling receptor for GDNF ligands.

### RET-independent signaling by GDNF and GFRα1 through the neural cell adhesion molecule NCAM

The search for GDNF receptors was motivated by the expectation that these could be targeted with small molecules which may someday become new therapies for Parkinson’s disease. Researchers were looking for sources of such receptors in primary cells and cell lines that showed binding activity and/or biological responses to GDNF. RET and GFRα1 were indeed discovered as a result of studies in kidney cells (Durbec et al. 1996), motoneuron cell lines (Trupp et al. 1996), retinal ganglion cells (Jing et al. 1996), and midbrain dopaminergic neurons (Treanor et al. 1996). After the storm caused by the RET and GFRα1 discoveries settled down, researchers were left with several cellular sources of GDNF-binding activity that could not be explained by either of the two receptors. Thus, for example, the neural precursor cell line RN33B as well as primary Schwann cells expressed GFRα1 but not RET and displayed high molecular weight species in chemical cross-linking experiments of ≈ 160 kDa (corresponding to a GDNF-binding moiety of ≈ 140 kDa) that were too small to be RET and too large to be dimers of GFRα1 (Paratcha et al. 2003; Trupp et al. 1999). In RN33B cells, a Src-like kinase activity was recovered from lipid rafts and GFRα1 immunoprecipitates that could be enhanced by stimulation with GDNF (Trupp et al. 1999). This activity could subsequently be attributed to Fyn kinase in both RN33B cells and Schwann cells, leading to activation of the CREB transcription factor (Paratcha et al. 2003; Trupp et al. 1999). Based on its molecular weight, downstream signaling and the type of cells where it could be found, one of us (G.P.) speculated that the mysterious p140 could be the 140 kDa isoform of the neural cell adhesion molecule NCAM. This was subsequently confirmed by immunoprecipitation of cross-linked complexes between GDNF and p140 with NCAM antibodies, as well as reconstitution of GDNF binding by transfection with NCAM cDNA in heterologous cells (Paratcha et al. 2003). These studies also established that although NCAM could associate with GDNF in the absence of GFRα1, both receptors were required to generate high-affinity-binding sites (K_D_ ≈ 1 nM) and elicit downstream signaling. Interestingly, other members of the GFRα (e.g., GFRα2 and 4) family could also mediate binding of their corresponding ligands (i.e., NTN and PSP) to NCAM (Paratcha et al. 2003). In addition to promoting high-affinity binding of GDNF ligands, the association of GFRα1 with NCAM prevented homophilic NCAM-NCAM interactions (Paratcha et al. 2003), illustrating the ability of GFRα1 to transform NCAM from a short-range cell adhesion molecule into a long-range signaling receptor for diffusible GDNF ligands. Subsequent structure/function studies identified the third immunoglobulin (Ig) domain of NCAM as the necessary and sufficient determinant for its interaction with GDNF (Nielsen et al. 2009; Sjöstrand et al. 2007) and established that GFRα1 blocks NCAM-mediated cell adhesion through its association with NCAM’s fourth Ig domain (Sjöstrand and Ibáñez 2008). To date, a myriad of in vitro and in vivo studies have provided a broad insight into the mechanisms through which RET-independent GDNF signaling through GFRα1 and NCAM regulates different cellular processes during nervous system function and development. Functions attributed to this alternative GDNF signaling system include proliferation (Bonafina et al. 2018), survival (Chao et al. 2003; Ilieva et al. 2019), migration (Paratcha et al. 2003, 2006; Wan and Too 2010), neurite outgrowth (Cao et al. 2008a; Chao et al. 2003; Irala et al. 2016; Nielsen et al. 2009; Paratcha et al. 2003), axon guidance (Charoy et al. 2012), and dendrite development and synapse formation (Irala et al. 2016; Ledda et al. 2007).

Early investigations using tissue explants from *Ret*- and *Ncam*-null mice pointed to the importance of GDNF signaling through GFRα1 and NCAM for migration of Schwann cells as well as neuroblasts in the rostral migratory stream, both of which express GFRα1 but not RET. GDNF-mediated chemoattraction of Schwann cells and olfactory neuron precursors could be abolished by NCAM-blocking antibodies or a null mutation in the *Ncam* locus (Paratcha et al. 2006; 2003). Moreover, *Gfra1* knock-out mice phenocopied abnormalities observed in the rostral migratory stream of *Ncam* knock-out mice (Paratcha et al. 2003). Later studies found that the effects of GFRα1 are restricted to precursor cells that give rise to all major classes of OB interneurons, as the receptor is later downregulated as these neurons mature (Zechel et al. 2018). Conditional ablation of GFRα1 in embryonic GABAergic cells recapitulated the cell losses previously observed in global *Gfra1* knock-outs at birth (Marks et al. 2012), revealing a requirement for the sustained generation and allocation of olfactory bulb interneurons. Conditional loss of GFRα1 in GABAergic precursors altered the migratory behavior of neuroblasts along the rostral migratory stream and affected their differentiation (Zechel et al. 2018), phenotypes that are all identical to those found in *Ncam* mutants (Chazal et al. 2000; Röckle and Hildebrandt 2016). Similarly, GDNF-induced Schwann cell migration was also shown to require binding to NCAM and activation of Fyn kinase (Paratcha et al. 2003; Zhou et al. 2003). In agreement with these findings, subsequent studies established that RET-independent GDNF signaling regulates Schwann cell function at pre-myelinated stages by activating the NCAM-Fyn-ERK1/2-CREB signaling pathway (Iwase, et al. 2005). Later studies used knock-down of GDNF receptors to provide additional evidence linking GDNF/NCAM signaling to migration of C6 glioma cells (Wan and Too 2010). It could also be shown that ablation of polysialic acid (PSA) chains from NCAM by enzymatic treatment interferred with GDNF-induced migration of TE671 skeletal muscle-derived cells (Conchonaud et al. 2007). In all these cases were the effects of GDNF on cell migration mediated by NCAM in a RET-independent manner.

A recent study demonstrated that GDNF signaling through GFRα1 antagonizes the effects of the mitogenic factor FGF2 in the proliferation and self-renewal of glutamatergic neural progenitor cells at embryonic stages of cortical development. Interestingly, these cortical progenitor cells lacked RET, and the inhibitory effects of GDNF were antagonized by function-blocking NCAM antibodies, supporting the role of GDNF/GFRα1 signaling via NCAMs in cortical neurogenesis (Bonafina et al. 2018). This finding is in line with previous work indicating that NCAM overexpression reduced fibroblast cell proliferation in response to FGF (Francavilla et al. 2007).

RET-independent GDNF signaling through GFRα1 and NCAM has been shown to have diverse effects on axonal growth and guidance in a variety of neuronal populations, including hippocampal, cortical, and dopaminergic neurons (Chao et al. 2003; Nielsen et al. 2009; Paratcha et al. 2003). By investigating GDNF expression in *gdnf-lacZ* reporter mice, Charoy and colleagues discovered a role for GDNF signaling through NCAM in the guidance of commissural axons of the spinal cord (Charoy et al. 2012). They found a prominent and restricted expression of GDNF in the floor plate, where commissural axons cross the midline. Intriguingly, however, GDNF did not function as a direct chemoattractant but instead enhanced responsiveness to the midline repellent Semaphorin3B (Sema3B) by blocking calpain1-mediated processing of the Sema3B signaling co-receptor Plexin-A1 on crossing axons (Charoy et al. 2012). Through genetic and in vitro experiments, this effect was found to be mediated by GFRα1 and NCAM independently of RET.

GDNF has been shown to have profound effects on synapse formation through GFRα1 and NCAM receptors independently of RET. This was first shown in hippocampal neurons, through the process of ligand-induced cell adhesion mentioned above. The effects on presynaptic maturation were abrogated in GFRα1 knock-out-mice and partially inhibited in animals deficient in NCAM, indicating that NCAM is a necessary component for presynaptic machinery assembly induced by GDNF (Ledda et al. 2007). On the postsynaptic side, it was later found that postsynaptic differentiation induced by GDNF could be abolished by knock-down of NCAM expression. This also abolished the ability of GDNF and GFRα1 to induce growth and complexity of dendritic arbors and spines in the postsynaptic neuron (Irala et al. 2016). More recently, it was shown that GDNF signaling through GFRα1 is also required for proper dendritic maturation and synaptic integration of adult-born granule hippocampal neurons in the dentate gyrus, as well as for correct spatial pattern separation memory in mice (Bonafina et al. 2019) (Fig. 1). Adult-born hippocampal granule cells express GFRα1 and NCAM but not RET, suggesting a RET-independent mechanism with NCAM as the main signaling receptor, although this remains to be formally proven. Together, these studies reinforced the importance of GDNF/GFRα1 signaling via NCAM for the establishment of hippocampal connectivity. Additional studies will be required to elucidate the contribution of the structural plasticity promoted by GDNF, GFRα1, and NCAM in hippocampal neurons to learning and memory processes.

Several studies have also investigated possible contributions of GDNF and NCAM signaling in different pathophysiological conditions, such as chronic pain, drug addiction, neurodegeneration, and epilepsy. GDNF signaling through NCAM was found to promote analgesia in a rat model of neuropathic pain by modulating nociceptive responses in peripheral neurons (Sakai et al. 2008a, b). In cultured dopaminergic neurons of the ventral tegmental area, GDNF induced activation of the NCAM-associated kinase FAK (focal adhesion kinase), leading to reduced morphological and functional neuroadaptative responses to chronic morphine (Li et al. 2014). Using the SH-SY5Y cell line, the same research group later found that the neuroprotective effects of GDNF on chemically induced neurotoxicity triggered by administration of 6OH-dopamine required NCAM translocation into lipid rafts (Li et al. 2017). These findings were in agreement with an early study indicating that the effects of GDNF on survival, outgrowth, and dopamine turnover of midbrain dopaminergic neurons could be antagonized by application of function-blocking antibodies against NCAM (Chao et al. 2003), as well as other studies highlighting the importance of NCAM for survival of dopaminergic neurons in cell culture (Ditlevsen et al. 2007) and dopaminergic neuron function in vivo (Xiao et al. 2009). Additional evidence also suggests that GDNF signaling via PSA-NCAM restricts seizure-induced hippocampal neurodegeneration and epileptogenesis through a mechanism involving FAK phosphorylation (Duveau and Fritschy 2010). Given the multiple roles of GDNF signaling through NCAM on structural plasticity of a variety of neurons, it is likely that many more pathophysiological processes will be found in which this signaling system is implicated.

The ability of GFRα1 to inhibit NCAM-mediated cell adhesion in a dose-dependent manner when either co-expressed with NCAM in the same cell or exogenously added as a soluble protein suggested the possibility that GFRα1 and NCAM could regulate nervous system development independently of the presence of GDNF (Paratcha et al. 2003). The first demonstration of the physiological importance of this effect in vivo was presented by a recent study showing that GFRα1 can function independently of GDNF and RET to control Purkinje cell migration in the developing cerebellum by counteracting NCAM function through direct binding (Sergaki and Ibáñez 2017). In the absence of GFRα1, Purkinje cell migration was delayed, but this effect could be alleviated by reducing genetically NCAM expression. GFRα1 interacted directly with NCAM in Purkinje cells of the developing cerebellum (Sergaki and Ibáñez 2017). It is possible that some of the effects observed in the rostral migratory stream of *Gfra1* null mutants, including abnormal migration and stream enlargement (Paratcha et al. 2003), may be due to its ability to keep NCAM-mediated cell adhesion in check among population of migratory cells. By limiting NCAM cell adhesion functions, GFRα1 may help to regulate cellular migration in many sites of the developing nervous system. Such function may also be ancient and evolutionary conserved. Drosophila GDNF receptor-like (DmGfrl) encodes a GPI-anchored membrane protein with similarities to GFRα1, and one study found a genetic interaction between the gene encoding DmGfrl and the Drosophila NCAM homolog FasII in the regulation of fertility in the flies (Kallijärvi et al. 2012). The two proteins were also found to associate biochemically (Kallijärvi et al. 2012), a result that is also reminiscent of the interaction between GFRα1 and mammalian NCAM. In the next section, we describe other possible GFRα co-receptors and mechanisms used by GDNF ligands independently of RET and NCAM.

### Other mechanisms of RET-independent signaling by GDNF ligands

Although the preferred paradigm for RET-independent signaling by GDNF ligands involves the presence of an alternative transmembrane receptor, such as NCAM, early on researchers had speculated with other mechanisms not requiring a transmembrane partner. These ideas emanated from the ability of GFRα1 to couple to Src family kinases and the association with both types of proteins with lipid rafts (Poteryaev et al. 1999; Trupp et al. 1999). A phenomenon termed monolayer coupling had been described in sphingomyelin bilayer systems (Schmidt et al. 1978) which was later proposed as a possible signaling mechanism for GPI-anchored receptors involving coupling between rafts in the outer leaflet with rafts in the inner leaflet (Brown and London 1998). It remains possible that Src family kinases might be concentrated and activated simply by partitioning into lipid rafts, without the need of physically binding GFRα1 directly. Moving forward, it will be important to carefully rule out the involvement of other possible transmembrane molecules, not only RET, before delving into alternative mechanisms to explain RET-independent signaling. In this regard, for example, it has been reported that GFRα1 is able to promote GDNF internalization in fibroblasts independently of RET (Vieira et al. 2003), but NCAM can be present in fibroblasts (Nakatani et al. 2006). Likewise, the reported effects of GFRα1 independently of RET on cisplatin-induced chemoresistance in osteosarcoma (Kim et al. 2017) could also have involved NCAM present in these cells (Ely and Knowles 2010).

A number of studies have described unexpected signaling and biological activities of GDNF through GFRα1 independently of either RET or NCAM in cortical GABAergic interneurons and their precursors from the medial ganglionic eminence (MGE). The first set of studies used cultured MGE cells and knock-out mice lacking either GFRα1, RET, or NCAM to show that GDNF and GFRα1 promote differentiation and tangential migration of cortical GABAergic neurons independently of RET or NCAM (Pozas and Ibáñez 2005). GDNF induced the GABAergic phenotype in cultured MGE precursors and promoted their morphological differentiation. In organotypic cultures, microbeads coated with GDNF could induce the migration of GABAergic neurons in the cortex. These effects were abolished in cultures derived from *Gfra1* mutant mice, but not in those derived from either *Ret* or *Ncam* mutants. In vivo, newborn knock-out mice lacking GDNF or GFRα1 showed a marked reduction in cortical GABAergic neurons, but no such losses could be detected in mice lacking RET or NCAM (Pozas and Ibáñez 2005). As a way to circumvent the early lethality of *Gfra1* null mutants, a second study used the so-called cis-only mutant mice that lack GFRα1 only in cells that do not express RET and which survive to adulthood (Enomoto et al. 2004). At birth, the cis-only mice phenocopied the specific loss of GABAergic interneurons in rostro- and caudolateral cortical regions previously seen in the null mutants (Canty et al. 2009). Surprisingly, the adult cortex of cis-only mice displayed a complete loss of parvalbumin-expressing GABAergic interneurons in discrete regions interspersed among areas of normal parvalbumin cell density. Consistent with deficits in cortical inhibitory activity, cis-only mice showed enhanced cortical excitability, increased sensitivity to epileptic seizure, and abnormal social behavior (Canty et al. 2009). As GFRα1 had been removed exclusively from neurons that did not express RET, the effects of GFRα1 on these cells were per definition RET independent. Since knock-out mice lacking NCAM did not show deficits in cortical GABAergic neurons (Pozas and Ibáñez 2005), it was speculated that GFRα1 contributed to the development of these cells by partnering with an unknown transmembrane receptor, or through alternative mechanisms, such as ligand-induced cell adhesion. More generally, the results suggested a role for GFRα1 in the allocation of parvalbumin interneurons to specific cortical areas. Since the areas mostly affected, namely, caudal and frontal cortices, are the regions furthest away from the birth place of these cells in the MGE, a general decrease in neuronal migration could in principle explain the specific deficits in those areas. Because mature GABAergic neurons no longer express GFRα1 once in the cortex, GFRα1 may function by directing the differentiation and migration of a distinct subpopulation of GABAergic precursors to specific cortical areas. Searching for possible molecular mechanisms of GFRα1 function in these cells, a later study employed different in vitro systems to test the effects of soluble GFRα1 presented exogenously in the differentiation and migration of MGE cells (Perrinjaquet et al. 2011). These studies revealed that exogenous GFRα1 supplied in soluble form to primary MGE cultures derived from mutant mice lacking GFRα1 could rescue the effects of the mutation, a result that is only compatible with the existence of a transmembrane signaling partner for the GDNF-GFRα1 complex in GABAergic neurons. In the same study, two candidate receptors previously implicated in GABAergic neuron development were tested, namely, the neuregulin receptor ErbB4 and the receptor for hepatocyte growth factor MET. The MET receptor was also an intriguing GDNF receptor candidate, as a previous study had shown that GDNF could promote tubulogenesis of GFRα1-expressing kidney cells by inducing Src-mediated phosphorylation and activation of MET (Popsueva et al. 2003). However, GDNF did not induce the activation of either ErbB4 or MET in GABAergic cells from the MGE nor did inhibition of either receptor impair GDNF activity in these cells (Perrinjaquet et al. 2011). Surprisingly, MET inhibition or knock-out increased the expression of GFRα1 in MGE cells and promoted their differentiation, uncovering an unexpected interplay between GDNF/GFRα1/MET signaling pathways in the early diversification of cortical GABAergic interneurons.

As mentioned earlier, due to their highly positively charged molecular surfaces, several members of the GDNF family interact strongly with heparin-like molecules, including heparan sulfate (Ibáñez et al., unpublished and (Bespalov et al. 2011). Interestingly, heparan sulfate proteoglycans (HSPG), such as members of the syndecan family, are transmembrane molecules profusely decorated by heparin sulfate moieties, and a study from the laboratory of Mart Saarma and colleagues showed that syndecan-3 in particular could bind GDNF and other members of the family independently of GFRα1 (Bespalov et al. 2011). Intriguingly, GDNF ligands needed to be immobilized to extracellular substrates to effectively bind to and induce cell spreading and neurite outgrowth via syndecan-3, suggesting a clustering effect was involved. It is also interesting to note that these researchers found that deletion of the gene encoding syndecan-3 diminished the effects of GDNF on cell migration of MGE-derived GABAergic cells, although the effect of the mutation showed statistical significance only at very high (100 μM) concentrations of GDNF (Bespalov et al. 2011), way above the range used in the studies discussed above, suggesting that a very high receptor occupancy, such as that afforded by extracellular matrix clustering, may be required for syndecan-3 to mediate GDNF activities in those cells.

Finally, it should be mentioned that a few studies have reported that members of the integrin family may also be involved in GDNF signaling across the plasma membrane, although in most cases, the participation of RET in the effects observed was not tested and thus it remains unclear to which extent integrins can meditate GDNF effects on their own. In midbrain dopaminergic neurons, some of the effects of GDNF on survival, neurite outgrowth, and signaling could be diminished by blocking antibodies against either integrin αv or integrin β1 (Cao et al. 2008b; Chao et al. 2003). In the latter study, GFRα1 could be immunoprecipitated together with integrin β1 from tissue homogenates of the substantia nigra, containing dopaminergic neurons, and this was increased in brains that received injections of GDNF (Cao et al. 2008b), although it was not reported whether comparable levels of integrin β1 were immunoprecipitated under the different conditions used in the experiments. Moreover, as dopaminergic neurons also express RET and NCAM, the precise role played by integrins in the effects observed remains unclear. Another study investigated effects of GDNF on explants of midgut extracted from wild-type and mice lacking integrin β1 and found that GDNF could still induce outgrowth from the enteric neurons lacking integrin β1 but could not function as a chemoattractant, as the neurons remained within the explant, in contrast to the wild-type explants (Breau et al. 2006). Again, as these neurons express high levels of GFRα receptors and RET, it is unclear whether integrin β1 can function independently to mediate GDNF effects on cell migration. Finally, it has also been reported that RET knock-down in mouse sensory neurons abolished some, but not all, effects of GDNF on those cells in vitro, particularly in the evoked release of the neuropeptide calcitonin gene-related peptide (CGRP) (Schmutzler et al. 2011). However, it was not investigated whether GFRα1 or NCAM were required. In summary, although integrin signaling may in some cases contribute to the biological effects of GDNF ligands, requirement does not mean sufficiency, and so it remains unclear whether integrins can on their own mediate GDNF activities independently of RET or other GDNF receptors.

## Conclusions

As it is evident from the research described in this paper, RET-independent signaling by GDNF ligands and GFRα receptors contributes to a wide variety of important biological activities and physiological processes (summarized in Table 1). Research into these mechanisms has not only provided explanations for intriguing functions but also allowed the discovery of previously unknown phenomena, such as ligand-induced cell adhesion. From its initial discovery at the end of the 1990’s, the field has moved on at increasing pace, benefitting from a wealth of new tools and reagents, not the least a wide range of lines of transgenic and targeted mice that allowed teasing out the different molecular components contributing to the functions of GDNF family ligands. We look forward to several more decades of exciting discoveries.

**Table 1:** Biological activities and physiological processes involving RET-independent signaling by GDNF ligands and GFRα receptors

Biological activity or process	Molecules implicated	References
Dendrite/axon outgrowth and complexity	GDNF, GFRα1, NCAM, HSPG	(Irala et al. 2016; Bonafina et al. 2019; Cao et al. 2008a; Chao et al. 2003; Nielsen et al. 2009; Paratcha et al. 2003)
Synapse formation	GDNF, GFRα1, NCAM	(Irala:2016df; Bonafina et al. 2019; Ledda et al. 2007)
Cell migration	GDNF, GFRα1, NCAM, HSPG	(Bespalov et al. 2011; Paratcha et al. 2003, 2006; Pozas and Ibáñez 2005; Sergaki and Ibáñez 2017; Wan and Too 2010; Zechel et al. 2018)
Cell survival	ART, GFRα3, NCAM GDNF, NCAM, Integrin αv	(Chao et al. 2003) (Ilieva et al. 2019)
Axon guidance	GDNF, GFRα1, NCAM, Sema3B	(Charoy et al. 2012; Ledda et al. 2002)
Neural precursor cell proliferation	GDNF, GFRα1, NCAM	(Bonafina et al. 2018)
